# Developmental evolution facilitates rapid adaptation

**DOI:** 10.1038/s41598-017-16229-0

**Published:** 2017-11-21

**Authors:** Hui Lin, Romas J. Kazlauskas, Michael Travisano

**Affiliations:** 1 BioTechnology Institute, University of Minnesota, St Paul, Minnesota 55108 USA; 2 Department of Biochemistry, Molecular Biology and Biophysics, University of Minnesota, St Paul, Minnesota 55108 USA; 3 Department of Ecology, Evolution and Behavior, University of Minnesota, St Paul, Minnesota 55108 USA

## Abstract

Developmental evolution has frequently been identified as a mode for rapid adaptation, but direct observations of the selective benefits and associated mechanisms of developmental evolution are necessarily challenging to obtain. Here we show rapid evolution of greatly increased rates of dispersal by developmental changes when populations experience stringent selection. Replicate populations of the filamentous fungus *Trichoderma citrinoviride* underwent 85 serial transfers, under conditions initially favoring growth but not dispersal. *T. citrinoviride* populations shifted away from multicellular growth toward increased dispersal by producing one thousand times more single-celled asexual conidial spores, three times sooner than the ancestral genotype. Conidia of selected lines also germinated fifty percent faster. Gene expression changed substantially between the ancestral and selected fungi, especially for spore production and growth, demonstrating rapid evolution of tight regulatory control for down-regulation of growth and up-regulation of conidia production between 18 and 24 hours of growth. These changes involved both developmentally fixed and plastic changes in gene expression, showing that complex developmental changes can serve as a mechanism for rapid adaptation.

## Introduction

The potential for rapid adaptation is a topic of increasingly broad interest. Given the pace of human induced environmental change, are populations and species capable of rapid Darwinian adaptation? This is especially relevant to species at risk, because smaller populations have fewer genetic variants, so adaptive evolution proceeds slowly or not at all^1^. Developmental evolution provides a potential mechanism for rapid adaptation, even for small populations. Dramatic phenotypic changes have been observed in studies of developmental mutants^2,3^, affecting growth and dispersal^4,5^. Many of the underlying genetic changes involved regulatory structures, such as the HOX gene cluster found in almost all metazoans and homeobox genes found in fungi, plants and animals. The hierarchical genetic regulatory control of developmental traits provides an avenue for rapid phenotypic evolution by few mutations^6^. Nevertheless, understanding the interplay between developmental and evolutionary processes has proven challenging, particularly at the microevolutionary scale.

**Figure 1:**
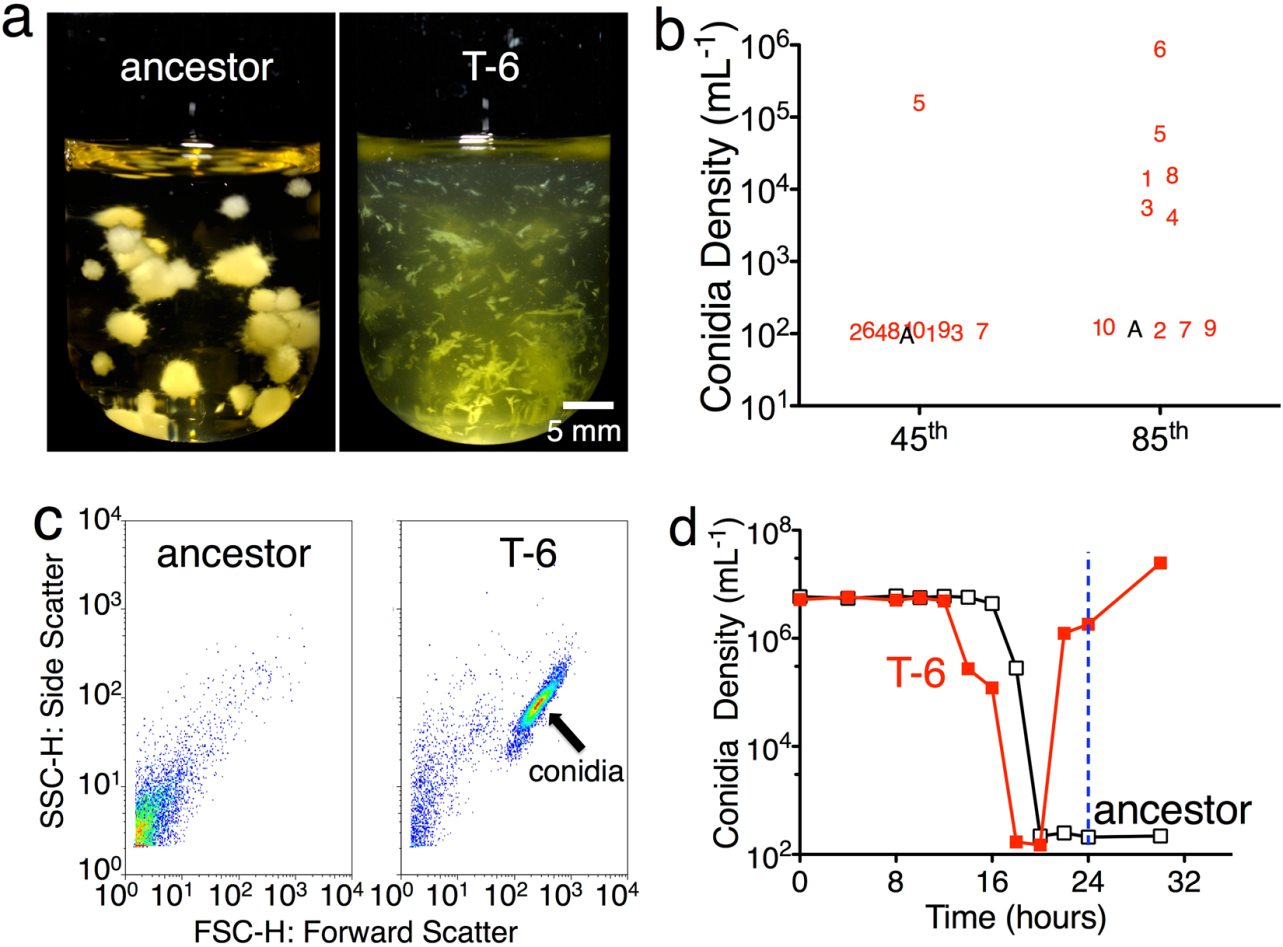
*T. citrinoviride* conidiation. (**a**) Ancestral *T. citrinovirde* grows as mycelia balls in malt extract media with no transferable conidia production in 30 hours (clear liquid), while selected *T. citrinovirde* T-6 strain produced large numbers of conidia (>10^6^ conidia per mL, green) in malt extract media, resulted in a cloudy liquid culture. (**b**) No fresh conidia production of the ancestral population (A) in 24 hours, while one of ten 45th derived populations (numbers) and six of ten 85th derived populations (numbers) produced large numbers (10^3^–10^6^ per mL) of conidia in liquid cultures without filter paper strips in 24 hours. (**c**) Flow cytometry dot plots show no conidia in the ancestor liquid cultures, but large numbers of conidia in the T-6 liquid cultures. (**d**) Upon transfer to fresh media, condia density declines sooner in T-6 than the ancestor, as a consequence of more rapid germination. Condia formation starts far sooner in T-6, yielding about 10^6^ conidia per mL in by 24 hours, while no conidia production occurs in the ancestor by that time.

To address these issues, we use selection on experimental populations of the filamentous fungus *Trichoderma citrinoviride* to determine the potential and basis for rapid adaptation. Experimental evolution studies can provide empirical validation and mechanistic support for theory involving evolutionary responses^7,8^. *T. citrinoviride* is a common soil fungus that grows rapidly spreading via hyphae and aerially dispersed asexual spores (conidia) under field conditions and when propagated on solid media^9^. Like many filamentous fungi, its growth is dramatically altered in submerged liquid culture^10^, in which asexual conidiation is suppressed and mycelia form large multicellular aggregates (Fig. 1a). These changes substantially limit dispersal. Populations rapidly decline to extinction within five daily serial transfers, because the liquid phase contains few, if any, propagules (cells or conidia) (Fig. 1a), even though *T. citrinoviride* grows rapidly in nutrient rich agitated liquid media.

To investigate the mechanisms of improved dispersal, we first explored culture conditions that select for the evolution of dispersal. Populations that were vigorously agitated to suspend and disperse the fungal biomass prior to transfer go extinct within ten passages similar to those without agitation. Addition of 3 mm glass balls to the culture media broke up multicellular masses, but also killed most cells which again led to population extinction. The multicellular masses were dispersed to near extinction levels of about 100 individuals per 0.1 mL at transfer, by addition of filter paper strips and vigorous agitation (Supplementary Fig. s1). Agitation with filter paper strips was essential, as seen in a control experiment. Ten replicate populations were propagated by daily serial passage for 85 passages under these near extinction conditions (growth media containing filter paper strips, gentle agitation prior to transfer). We expected these minimal survival conditions to select for increased dispersal, and if there were evolutionary responses, that they would involve changes in growth form or spore production.

## Results

We observed substantial reductions in the susceptibility to extinction after selection. Selected populations persist at over 300-fold higher transfer population sizes than the ancestral genotype in the selected environment (ANOVA planned contrast *F*_*1,11*_ = 15.211, *p* = 0.0025). Even under the conditions that lack the filter paper and in which the ancestral genotype rapidly goes extinct, the risk of extinction was eliminated in six of ten derived populations (Fig. 1b). These results indicate that *T. citrinoviride* rapidly evolved and the derived populations could now persist in previously unsuitable conditions.

Identifying the mechanisms by which adaptation proceeds is crucial for determining its generality across biological systems. The lowered risk of extinction in the *T. citrinoviride* populations could have involved increases in total growth, per capita dispersal, or some combination of both. We observed no increase in net growth over the course of selection, as both total biomass and hyphal biomass declined across all the replicates (although the T-9 population has similar biomass with the ancestor) following selection (respectively, adj. *R* ^2^ = 0.96, *F*_*1,1*2_ = 77.79, *p* < 0.0001; adj. *R* ^2^ = 0.96 and *F*_*1,12*_ = 87.29, *p* < 0.0001: Supplementary Fig. s2). In contrast, the liquid fractions of the derived populations have abundant suspended material, propagules, hyphae or conidia depending upon the replicate (Fig. 1b, Supplementary Fig. s1). The derived populations differed substantially in amount of suspended microscopic material (*F*_*9,10*_ = 5.08, *p* = 0.0091), and counts of the suspended biomass are strongly correlated with population size in the suspended media (*r* = 0.90, *p* < 0.0003) (Supplementary Fig. s1). Three characteristics indicate that the phenotypic differences are most likely genetic, not epigenetic. First, the differences evolved gradually over the course of selection. Increased production of conidia required 45 transfers, ~300 generations, for one population and 85 transfers, ~560 generations, for five more populations. Second, the phenotypes differed from each other across replicates. Not all populations showed increased conidia production; some showed increased numbers of small hyphae in suspension. Third, the phenotypic differences among the selected populations and the ancestor, were stable and heritable. Storage, either on plates or −80 °C, did not affect the evolved differences among populations in the selected environment, which allowed multiple replicates to assayed over time and high degrees of statistical confidence. The gradual evolution, diverse phenotypes and stable phenotypic differences are all characteristic of genetic, rather than epigenetic, changes.

**Figure 2:**
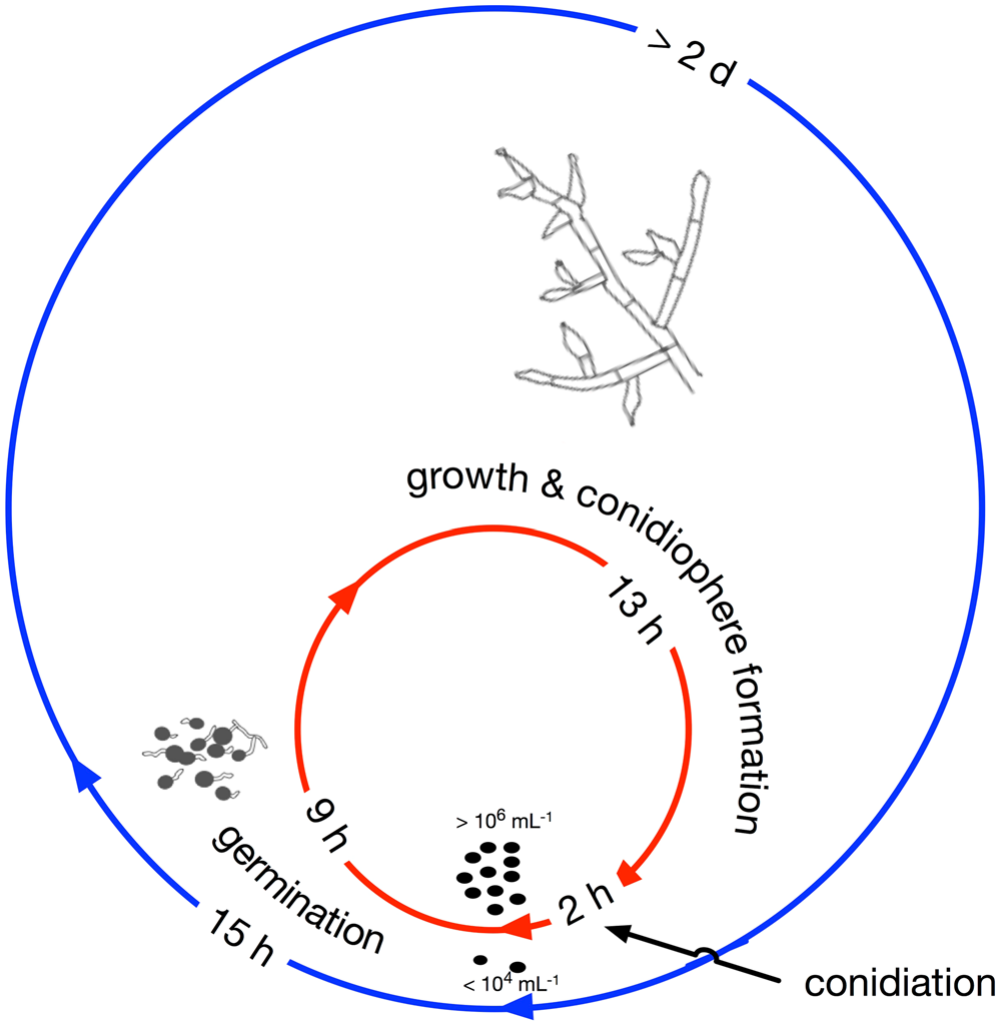
Ancestor (outer) versus T-6 (inner) life cycle (Supplementary Fig. s4). (1) The ancestor (blue) requires 15 hours for 50% conidia germination, while T-6 (red) requires 9 hours to reach 50% germination (Supplementary Fig. s5); (2) T-6 has developed conidiophores after 22 hours and produces 10^6^ conidia per mL in 24 hours, while ancestor requires over 2 days for the conidiophore formation and produces no more than 10^4^ conidia per mL.

To further understand the basis of the increased propagule number, we focused investigation on population T-6. This population is one of the six conidia-producing populations, and produces the largest numbers of conidia with only few free hyphae (Fig. 1a,b,c and Supplementary Fig. s1, s2), and it also grew faster, and produced conidia earlier that the ancestor on the malt extract agar plate (Supplementary Fig. s3). We focused on population T-6 because of fewer morphological differences with the ancestor, which could lead to better clarity in disentangling expression differences. The large amounts of conidia give the media a green color and indicate that suppression of conidiation due to submerged growth had been alleviated. Relative to the ancestor, the T-6 genotype produces conidia 50–67% faster (24 versus 48–72 hours, Fig. 2 and Supplementary Fig. s4) and generates conidia at 1000-fold greater numbers (10^6^ versus 10^3^ mL^−1^, Figs 1d and 2). This production is faster and greater than the ancestor on solid substrates, the ancestral preferred environment. In addition, in the T-6 genotype conidia germinated 40% faster than the ancestor (9 versus 15 hours, Fig. 2 and Supplementary Fig. s5a). These large changes in the extent and timing of conidia germination and production demonstrate that dramatic developmental changes readily evolve during experimental evolution in response to strong selection.

**Figure 3:**
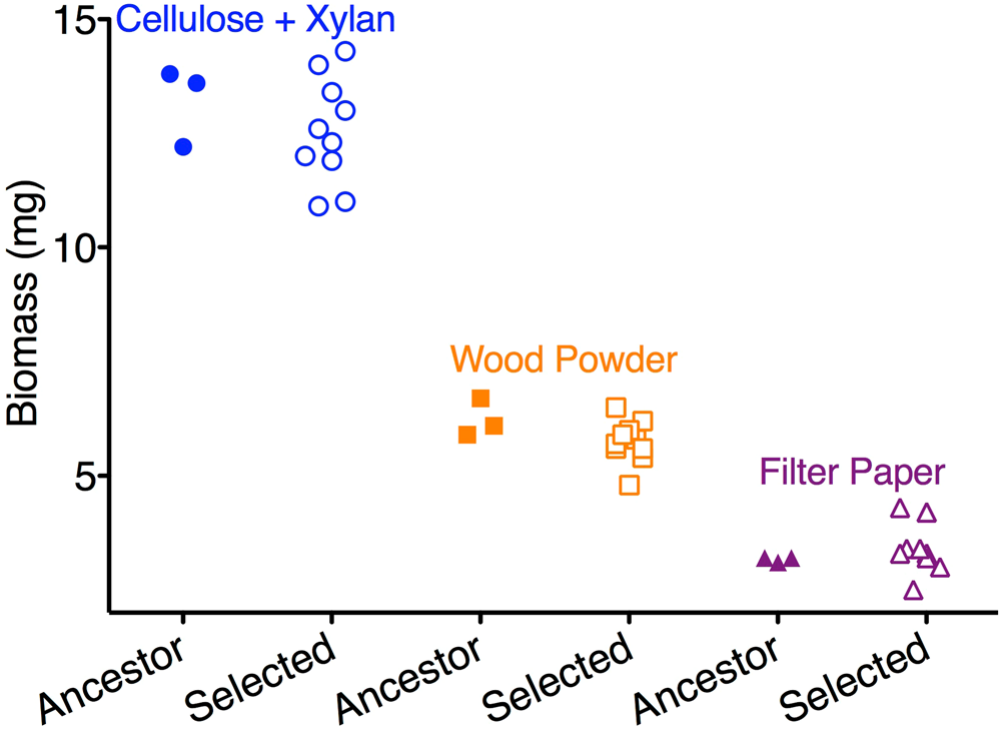
Mycelium growth in malt extract and novel nutrient conditions. The derived populations had similar or less than half amount of the biomass of the ancestor, while all ancestral and ten derived *T. citrinoviride* populations had same relative mycelium growth (Supplementary Fig. s6) in cellulose and xylan, wood powder, and filter paper in mineral salts media.

To test the environmental specificity of the developmental changes, we measured the growth and conidia production of derived *T. citrinoviride* under aqueous growth without malt extract, containing only one of the following: powdered cellulose/xylan, wood powder, or fibrous cellulose (filter paper) in mineral salts solution^11^. The three novel media differed across a four-fold range in the amount of growth supported (ANOVA adj. *R* ^*2*^ = 0.98, *F*_*2,20*_ = 498, *p* < 10^−17^), with the cellulose/xylan mixture, wood powder and fibrous cellulose supporting 60%, 80% and 90% less growth than the selected environment, respectively (Supplementary Fig. s6). Relative differences in growth between the ancestral and all ten derived populations remained constant, regardless of overall growth supported by each media (ANOVA planned contrast *F*_*1,6*_ = 1.70, *p* = 0.24) (Fig. 3). However, no conidia were observed in any of the novel nutrient conditions, none of which contained malt extract, demonstrating the evolution of highly condition-dependent changes in conidia production pathways and the developmental decoupling of this trait from growth. These results indicate that the developmental changes observed can be surprisingly limited to the specific environmental conditions in which they evolved.

There are at least two potential causes for the observed environmental specificity: the absence of developmental cues and limiting resources. We tested for this by measuring conidial production of the derived genotypes across a gradient of malt extract, and observed that malt extract is both a cue and a limiting resource. Addition of even a modest amount of malt extract (7.5 g/L) in cellulose/xylan powder media led to dramatic increases in conidia production (3.8 × 10^5^ conidia per mL), a response consistent with a cue. Focusing specifically on the T-6 genotype, we similarly observed large increases of conidia production with increasing concentrations of malt extract, but little effect on overall biomass across most of the range of malt extract (Supplementary Fig. s7). To better elucidate the effects of biomass on conidia production, we measured the biomass and conidia production of T-6 during growth in YPD (yeast-peptone-dextrose). We observed no detectable conidia, even after three days of growth, despite the largest biomass in any media (33.2 mg), confirming that reduced conidia production is not simply a consequence of biomass production. Thus, conidia production necessarily requires resources, as expected with the trade-off with hyphal biomass, and the expression of costly traits is cued to the resources involved for their evolution. Notably, these interactions are the consequence of the selection experiment. While conidia production requires malt extract media in the selected populations, malt extract does not induce abundant conidia production in the ancestral genotype. Indeed, the ancestral genotype of *T. citrinovride* was maintained on malt extract agar plates for years, but did not evolve abundant conidia production. Only the serially selected populations produced abundant conidia.

**Figure 4:**
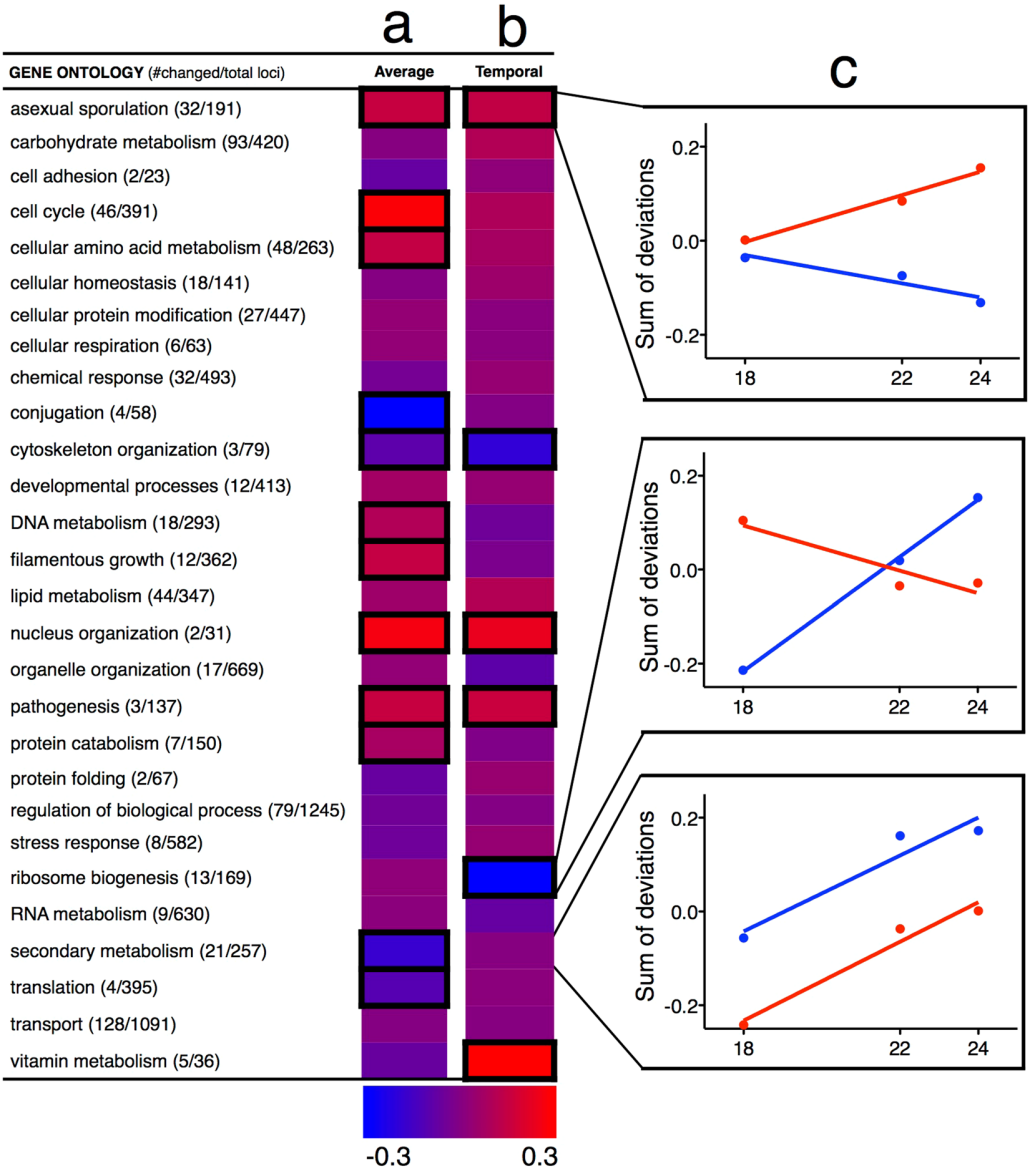
Functional analysis of transcription level evolution. (**a**) Twelve of the 28 GO groups (framed) have statistically significant directional changes in expression between ancestor and the derived T-6 genotype. Color indicates higher relative expression between the ancestor or T-6, blue and red, respectively, with the maximal 32% difference in expression. Eight GO groups have greater expression in T-6, while four GO groups were down-regulated. (**b**) Six GO groups have temporal changes in gene expression between the ancestor and T-6. In four cases, increasing expression from 18 to 24 hours in T-6 relative to the ancestor, and in two cases declining. Color indicates differences in relative expression from 18 to 24 hours between the ancestor and T-6, blue and red, respectively, with a maximal 10% change in temporal expression. (**c**) GO grouped loci expression in the process from conidia germination (18 hours) to conidiophore development (22 hours) to conidia production (24 hours). The Y axis shows deviations from average expression, evaluated across all three time points (18, 22, & 24) over all loci within a GO term, for both evolved and ancestral genotypes. Color indicates expression for the ancestral (blue) and T-6 (red) genotypes. Transcriptional evolution was complex and involved diverse locus specific evolutionary responses.

To identify the molecular basis for developmental evolution, we determined the transcriptional changes underlying the increased conidia production. mRNA of the ancestral and derived conidia producer (T-6) was sequenced at 18, 22, and 24 hours post transfer to fresh media, timepoints at which the selected genotype completes conidia germination, initiates conidiophore development and produces conidia, respectively (Supplementary Fig. s4). Comparisons at each time point show that the ancestor and T-6 transcription levels differ by more than two-fold for 1715 of total 9737 loci, for at least one or more of the three sample times (Supplementary Fig. s8). Sequences were annotated using blast queries of the *Emericella nidulans* (anamorph *Aspergillus nidulans*) protein sequence database, the closest related species with well characterized protein functions. 695 annotated loci could be grouped based on assignments from the *A. nidulans* gene ontology (GO)^12^. A high proportion of the variation in transcription levels (89%) is explained by an ANOVA analysis using GO assignments to group loci (ANOVA adj. R^2^ = 0.89, *F*_*111,56*_ = 13.06, *p* < 0.0001). We observed large scale restructuring of gene expression over the course of selection, with statistically significant directional changes in expression in 12 of the 28 GO groups (Fig. 4a). Adaptation to the selective conditions did not occur by uniformly unidirectional changes, as expression in the derived T-6 genotype was greater in eight functional groups and lower in four. As anticipated, expression of loci tightly associated with conidia production (asexual sporulation) or required for conidia production (cell cycle, DNA metabolism, and nucleus organization) increased, while expression of loci having little utility in the selective environment (conjugation and secondary metabolism) decreased. The *brlA*, *abaA* and *wetA* are the central regulators in the asexual conidiation process^13^, and the RNA-seq data showed higher transcription of *wetA* and *abaA* in T-6 than the ancestor, T-6 had over 20-fold higher expression of *wetA* and *abaA* at 22-hour and 24-hour (Supplementary Table s1). Surprisingly, no changes were observed in stress associated gene expression, as stressful conditions can lead to simplified conidia production^14^. Other changes in expression (e.g., enhanced for cellular amino acid metabolism and reduced for translation) are less directly attributable to the qualitative responses in the selective environment, reflecting potential contributions from drift^15^, pleiotropy^16^, or quantitative fine-tuning during selection. These different directional responses occurred within 85 bouts of selection of at least 6.6 generations per bout (~ = log_2_^100^) or approximately 561 generations, demonstrating the potential for rapid large scale evolution for gene expression.

Even finer scale evolutionary changes in gene expression were observed in six GO functional groups. The derived T-6 genotype has distinct patterns of gene expression over the time course from 18, 22 to 24 hours, unseen in the ancestral genotype (Fig. 4b). Increasing expression from 18 to 24 hours is observed for four of the GO groups in the T-6 genotype relative to the ancestor. In contrast, decreasing expression is observed for two GO groups, demonstrating divergent evolutionary responses in the changes in temporal expression. These changes are largely consistent with prior expectations. Expression of loci associated with asexual reproduction increases from 18–24 hours in the derived genotype (Supplementary Fig. s8), but expression of ribosome biogenesis loci declines as conidia production increases (Fig. 4c). These transcriptomic changes indicate a broad scale retiming of gene expression for growth, genome replication and protein production necessary for conidia production.

## Discussion

The optimal solution for most evolved populations is a combination of propagules that includes conidia and broken hyphae. All replicates evolved hyphal fragmentation, including those with abundant conidia production. The model below shows that the combination of hyphal fragmentation and conidia production maximizes the number of propagules. The model posits that hyphae grow exponentially at rate r_h_ (time^−1^), then switch to conidia production at time t_c_. Some fraction of the hyphae, f (f varies between 0 and 1), fragment to form suspended particles. P_0_ is the initial number of propagules upon transfer to fresh media and H_T_ is the total number of fragmented and non-fragmented hyphae. Equation 1 yields the total number of suspended hyphal particles, f · H_T_.

1 $${\rm{f}}\cdot {{\rm{H}}}_{{\rm{T}}}=f\,\cdot \,{{\rm{P}}}_{0}{{\rm{e}}}^{{\rm{rhtc}}}$$

The number of conidia, CT, increase linearly according to the conidia production rate, r_c_, the number of total hyphae, H_T_, and the time between the onset of conidia production t_c_ and the time between transfer to fresh media, T, eq. 2. These assumptions are consistent with literature and our observations.

2 $${{\rm{C}}}_{{\rm{T}}}={{\rm{r}}}_{{\rm{c}}}{{\rm{H}}}_{{\rm{T}}}(T\,-\,{{\rm{t}}}_{{\rm{c}}})$$

The total number of propagules transferred, P_T_, is the sum suspended hyphal particles, f · H_T_, and the number of conidia, C_T_.

3 $${{\rm{P}}}_{{\rm{T}}}={\rm{f}}\cdot {{\rm{H}}}_{{\rm{T}}}+{{\rm{C}}}_{{\rm{T}}}$$

Solving for the root of dP_T_/dt_c_ reveals the onset time at which the number of propagules reaches a maximum, eq. 4.

4 $${{\rm{t}}}_{{\rm{c}}}(\text{for}\,{\rm{\max }}\,{{\rm{P}}}_{{\rm{T}}})={\rm{T}}+{f/r}_{{\rm{c}}}-{1/r}_{{\rm{h}}}$$

This model predicts the evolution of both conidia and hyphae propagules under a broad range of conditions. Equation 4 shows the importance that extent of fragmentation (f) has on the conidial production. In the absence of fragmentation (f = 0), conidia are the only mechanism for propagule formation. The optimal onset time of conidia production will maximize the amount of hyphae biomass to make conidia, but leave time for conidia to form. The model predicts optimal onset time depends inversely upon hyphal growth rate, because slower growing hyphae produce a smaller hyphal biomass than more rapidly growing hyphae over the same amount of time. When there is no fragmentation (f = 0), the rate of conidia production (r_c_) does not affect the optimal timing t_c_, as it does not affect hyphal biomass or the time remaining for conidia production.

**Figure 5:**
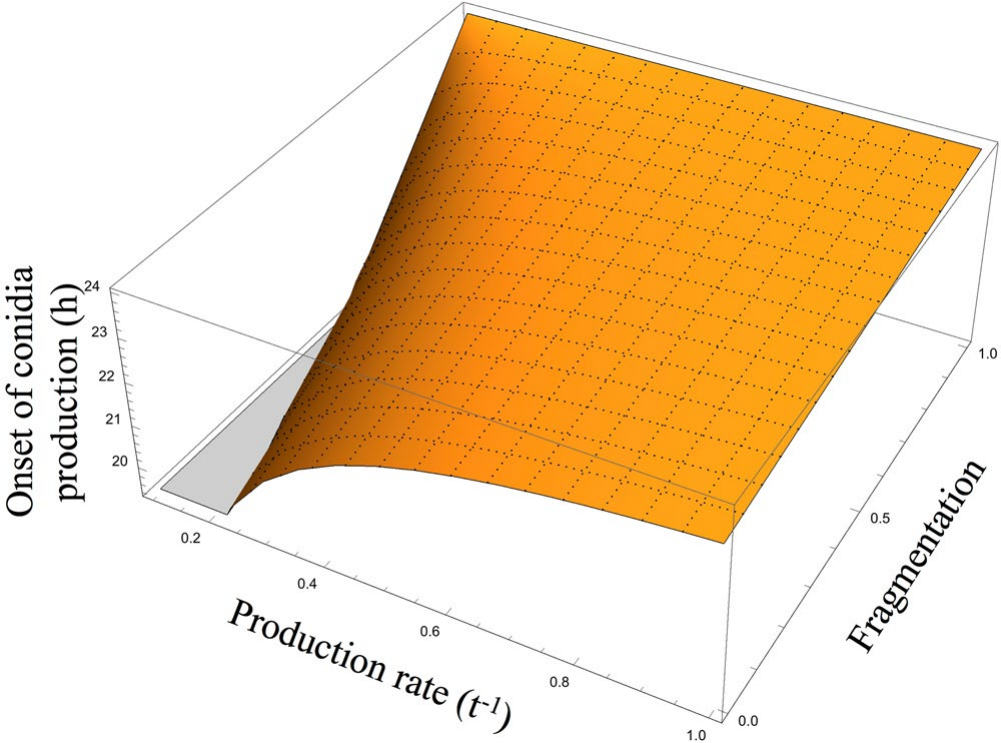
Predicted time for onset of conidia production, in a 24 hour transfer regime, as a function of production rates and hyphae fragmentation. This case assumes equal rates for production of conidia and hyphae. Almost all conditions favor conidia production. Increases in production rates and hyphal fragmentation favor later onset.

For intermediate hyphal fragmentation (0 < f < 1), we consider the conservative case where r_c_ = r_h_. The model predicts formation of conidia in most cases and a late onset of conidia formation as observed in some replicates (Fig. 5). The late onset greatly increases the number of hyphal fragments, and thereby the number of propagules at transfer. In practice, conidia production rates are higher than the rates of hyphal growth, increasing the relative benefits of conidia production.

Only in one case - complete hyphal fragmentation (f = 1) – does the model predict no conidial reproduction. In this case, exponential hyphal growth and fragmentation produces a greater number of propagules than a switch to conidia production with a linear growth rate (as reflected in the upper edge of the figure). Complete fragmentation corresponds to planktonic yeast-like growth, not filamentous, and would likely involve costly physiological changes.

Rapid evolution can readily proceed by changes in development. Previously, we demonstrated rapid evolution of multicellularity in two divergent species of initially single-cell yeasts under strong settling selection^17,18^. In both species, multicellular forms arose by developmental changes, which were due to mutations in a transcription factor in one of the species^19^, consistent with a well-known mode of developmental evolution^5^. Our current results demonstrate the potential for developmental evolution to modify multicellular phenotypes in the opposing direction, selection for improved dispersal led to the rapid evolution of increased unicellular growth stages. A large number of functional traits evolved over the course of selection, which could be clearly identified using Gene Ontology groupings. These changes were consistent with the developmental evolution observed in the populations, and took two modes. Twelve fixed functional differences were observed between the ancestral and derived genotypes, as identified by mean differences in expressed mRNA. Some of these fixed differences involved changes in development (e.g. increased expression of loci associated with cell cycle), although others (e.g., amino acid metabolism) suggest changes in non-developmental traits were also involved. Importantly, we also observed changes in the timing of expression between the derived genotype and ancestor between 18 and 24 hours. These plastic functional differences were identified by changes in temporal mRNA expression, all of which necessarily reflect developmental changes. These developmental evolutionary changes, fixed and plastic, indicate the importance of both modes of adaptation in response to environmental challenges, supporting Eco-Evo-Devo perspectives in understanding rapid evolutionary responses^20,21^.

The developmental patterns differ from those predicted by simple cooption of a preexisting mode of environmental induction for rapid conidiation. Most ascomycete fungi under stressful physical and/or chemical conditions largely or completely eliminate hyphal growth and undergo microcycle conidiation producing many conidia^22^. In contrast, our derived populations show both rapid hyphal growth and rapid conidiation. The increased conidia production is cued by resource availability, and does not involve changes in expression of stress response genes. The contrast between known patterns of conidiation and those observed in our study indicates that phenotypically variable traits are likely to be even more plastic and available for adaptation than currently anticipated. The selective benefits developmental evolution are nevertheless shaped by the ecological conditions and context. Our observations of strong environmental specificity of the developmental adaptations, involving both limiting resources and cues, reinforces the importance of both fixed and plastic developmental evolutionary and the ecological context in which they evolved.

## Material and Methods

*Trichoderma viride* strain FP-102208 was obtained from Forest Products Lab, WI, and DNA sequencing of the internal transcribed spacer identified this strain as *Trichoderma citrinoviride* ^23^. The RNA sequencing experiment was done in duplicate, and all the other experiments were done in triplicate. Conidia in liquid samples were counted by using a FACSCalibur flow cytometer (Becton Dickinson Sciences). A total of 50,000 flow cytometry events or 100 seconds were acquired per sample and gated in scatter diagrams of visible light (forward and side scatter) to exclude small debris. The density of conidia in the culture was calculated as counts per second per flow rate (0.2 uL/s) after correction for background counts. All the statistic analysis was run on JMP 12.

### Selection experiment

A malt extract plate (15 g malt extract, BD Difco, and 15 g agar in 1 L water) was inoculated with a loop (10 uL) of ancestral *T. citrinoviride* (*T. viride* strain FP-102208) conidia from frozen (−80 °C) stock and cultured at 28 °C for 5 days. A conidia suspension was prepared by flooding the plate with sterile water (10 mL) and carefully scraping the hyphae with a sterile inoculation loop. Ten replicates of sterile malt extract media (10 mL, 15 g malt extract in 1 L water) containing 10–15 strips of filter paper (2 × 30 mm, filter paper 415, VWR, UK) in test tubes (25 × 150 mm) were inoculated with this conidia suspension to yield 5 × 10^6^ conidia per mL and incubated at 28 °C and 240 rpm under ambient laboratory lighting. The test tubes were shaken gently to suspend small particles, but no attempt was made to break up larger clumps. An aliquot (0.1 mL) was removed using a manual adjustable volume pipette (volume 0.1–1.0 mL, Eppendorf) with a plastic 1-mL tip, which has an opening of ~0.07 mm. The removed volume was transferred to fresh malt extract media (10 mL) containing 10–15 strips filter paper (approximately 2 × 30 mm) in test tubes (25 × 150 mm) each day. After every 40 transfers, samples were stored for further analysis. For storage, the cultures were grown for 7 days in malt extract media to produce conidia, mixed with sterile glycerol (final glycerol concentration of 20%) and stored at −80 °C.

### Conidia formation and amount of biomass during growth

Malt extract media (10 mL) in test tubes (25 × 150 mm) was inoculated with conidia suspensions of ancestral or selected *T. citrinoviride* populations to a final conidia concentration of 5 × 10^6^ conidia per mL and incubated at 28 °C and 240 rpm. Aliquots (0.2 mL) were transferred to micro centrifuge tubes (1.5 mL) at 24, 36, 48 and 72 hours and kept on ice, and then the conidia concentrations were counted using a FACSCalibur flow cytometer. To measure the amount of hyphal biomass in ancestor and selected population cultures, malt extract media (10 mL) in test tubes were inoculated as above and incubated for 1 day at 28 °C and 240 rpm. The cultures were filtered through weighed four layers of cheese cloth and washed by MilliQ water (10 mL × 2), the residues were transferred into weighed glass vials, dried at 105 °C overnight and weighed. To measure the amount of conidia, the filtrate was transferred to weighed conical tubes, centrifuged at 13000 rpm for 10 min, then the supernatant was discarded and the residue was dried at 105 °C overnight and weighed.

### Numbers of live particles suspended in the culture media

Malt extract media (10 mL) in test tubes was inoculated with conidia suspensions of ancestral or selected *T. citrinoviride* populations to a final conidia density of 5 × 10^6^ conidia per mL and incubated at 28 °C and 240 rpm. High background counts due to loose fibers from the filter paper by vigorous agitation prevented estimation of conidia in the selective environment. An aliquot (0.1 mL) was transferred daily to fresh malt extract media containing strips. After 3 transfers, 0.1 mL of the liquid culture was transferred to 0.9 mL malt extract media, and then further serially diluted 10-fold for up to 10^8^-fold dilution. All of the serial dilution samples were cultured at 28 °C and 240 rpm for 2 days. Growth pellets in the cultures showed positive growth. The filter paper strips were removed from the test tubes, and the supernatant was spun at 13000 rpm for 10 min, and the amount of hyphal biomass in the residues were measured by HPLC based on the extracted ergosterol^24^.

### RNA isolation and sequencing

Malt extract media (10 mL) in test tubes were inoculated in triplicate with conidia (final conidia concentration of 5 × 10^6^ conidia per mL) from 5 day old cultures of ancestral *T. citrinoviride* and derived *T. citrinoviride* T-6 on agar plates. The cultures were incubated at 28 °C and 240 rpm. The culture from one tube of both ancestor and T-6 were transferred to a sterile 15 mL conical tube at 18, 22 and 24 hours, and centrifuged at 8000 rpm, 4 °C, 20 min. The supernatant was discarded and the remaining mycelia were resuspended in RNAlater solution (5 mL, Sigma) following the manufacturer’s protocol, and then centrifuged at 8000 rpm, 4 °C, 20 min. The supernatant was again discarded and the remaining mycelia were kept in −80 °C freezer, and then used for RNA isolation by using RNeasy Plant Mini Kit (Qiagen, Cat. No. 74903) following the manufacturer’s protocols. Total RNA was used as template for the construction of a cDNA library (Nominal size is 200 bp of +/−5% deviation by gel selection), using paired end reads with lengths of 101 bp on the Illumina Next-generation sequencing HiSeq2000 with duplicates of each sample and in the same lane by University of Minnesota Genomics Center.

### Transcriptome assembly, expression analysis and gene ontology analysis

Transcriptome assembly and differential expression analysis of the RNA-seq library were performed in Galaxy (https://galaxy.msi.umn.edu/). Sequencing reads were assigned to their most likely locus of origin by alignment to the unmasked assembled *genome* of *T. citrinoviride* v4 (Joint Genome Institute, http://genome.jgi.doe.gov/Trici4/Trici4.home.html) using Tophat2 (version 0.6; http://ccb.jhu.edu/software/tophat/index.shtml)^25^ with the filtered gene annotation (JGI, Trici4_GeneCatalog_genes_20131015.gff). The mapping rate was >80% for all libraries. The differential expression of individual genes was identified by Cuffdiff (version 0.0.6) (http://cufflinks.cbcb.umd.edu/) using geometric normalization^26^. Expression was computed as the normalized value of fragments per kilobase of feature sequence per million fragments mapped, or FPKM. Loci of ancestor and T-6 that showed at least a tw o-fold change in expression (log_2_[fold change] > 1) were further analyzed.

To identify the function of the proteins encoded by the extracted loci, their sequences were aligned to the local STRING protein sequence database of *Aspergillus nidulans* (*Emericella nidulans*) (162425.protein.sequences.v10, http://string-db.org/)^27^ in BlastStation Local using BLAST (Basic Local Alignment Search Tool). These annotated proteins were submitted to AspGD GO-Slim: Process database (http://www.aspergillusgenome.org/cgi-bin/GO/goTermMapper)^14^ to create a gene ontology (GO) Slim Mapper maps.

For statistical analysis, individual expression reads were log transformed, log(X + 1), to normalize the distribution of expression values. On a per locus basis, reads were divided by the summed expression across the three time points and both ancestral and derived genotypes. This normalization preserves the directionality of expression changes (positive or negative) for each locus. ANOVA of the scaled results reflects relative, and not absolute, changes in expression across loci.

### Growth and conidation of T. citrinoviride T-6 in new conditions

Cellulose (10 g/L) and xylan (5 g/L) in mineral salt solution (10 mL), wood powder (15 g/L) in mineral salt solution (10 mL)), or filter paper (10 g/L, strips (~30 strips measuring 1 × 15 mm) in mineral salt solution (10 mL) in test tubes were inoculated (5 × 10^6^ conidia per mL) with 5 days old T-6 conidia suspension. The cultures were incubated at 28 °C and 240 rpm. An aliquot of 0.2 mL was collected at day 1, day 2 and day 3, and the conidia in the cultures were counted by flow cytometer. After 3 days, all the cultures were transferred into 15 mL conical tubes, centrifuged at 10000 rpm and 4 °C for 20 min to harvest the total biomass. Then the amount of mycelia was measured by extracting ergosterol from the mycelia and quantification the amount by HPLC^24^.

Cellulose (10 g/L) and xylan (5 g/L) in mineral salt solution (10 mL) contained 7.5 g/L malt extract, or malt extract media (10 mL) with the malt extract concentration of 3.75, 7.5, 15, 30, 45 g/L in test tubes were inoculated (5 × 10^6^ conidia per mL) with 5 days old T-6 conidia suspension. The cultures were incubated at 28 °C and 240 rpm for 24 h. The cultures were collected and the conidia were counted using flow cytometry.

Yeast extract (10 g/L), peptone (20 g/L) and glucose (20 g/L) in test tubes were inoculated (5 × 106 conidia per mL) with 5 days old T-6 conidia suspension. The cultures were incubated at 28 °C and 240 rpm for 24 h. The cultures were collected and the conidia were counted using flow cytometry

### Data accessibility

Supporting data have been included in the supplementary. RNA-seq data have been deposited in the Sequence Read Archive (SRA) under Bioproject accession PRJNA304029.

## Electronic supplementary material

Supplemental Information

## References

[1] Lanfear R, Kokko H, Eyre-Walker A (2014). Population size and the rate of evolution. Trends Ecol Evol.

[2] Stern DL, Orgogozo V (2008). The loci of evolution: how predictable is genetic evolution?. Evolution.

[3] Murren CJ (2015). Constraints on the evolution of phenotypic plasticity: limits and costs of phenotype and plasticity. Heredity.

[4] Stern DL (2000). Perspective: Evolutionary developmental biology and the problem of variation. Evolution.

[5] Carroll SB (2008). Evo-devo and an expanding evolutionary synthesis: a genetic theory of morphological evolution. Cell.

[6] Erwin DH, Davidson EH (2009). The evolution of hierarchical gene regulatory networks. Nat Rev Genet.

[7] Lenski RE, Travisano M (1994). Dynamics of adaptation and diversification: a 10,000-generation experiment with bacterial populations. Proc Natl Acad Sci USA.

[8] Kawecki TJ (2012). Experimental evolution. Trends Ecol Evol.

[9] Harman GE, Howell CR, Viterbo A, Chet I, Lorito M (2004). Trichoderma species–opportunistic, avirulent plant symbionts. Nat Rev Microbiol.

[10] Gibbs PA, Seviour RJ, Schmid F (2000). Growth of filamentous fungi in submerged culture: problems and possible solutions. Crit Rev Biotechnol.

[11] Lin H, Travisano M, Kazlauskas RJ (2016). Experimental Evolution of Trichoderma citrinoviride for Faster Deconstruction of Cellulose. PLoS ONE.

[12] Arnaud MB (2012). The Aspergillus Genome Database (AspGD): recent developments in comprehensive multispecies curation, comparative genomics and community resources. Nucleic Acids Res.

[13] de Vries RP (2017). Comparative genomics reveals high biological diversity and specific adaptations in the industrially and medically important fungal genus Aspergillus. Genome Biol.

[14] Jung B, Kim S, Lee J (2014). Microcyle conidiation in filamentous fungi. Mycobiology.

[15] Whitehead A, Crawford DL (2006). Neutral and adaptive variation in gene expression. Proc Natl Acad Sci USA.

[16] Cheverud JM (1996). Developmental integration and the evolution of pleiotropy. Am Zool.

[17] Ratcliff WC, Denison RF, Borrello M, Travisano M (2012). Experimental evolution of multicellularity. Proc Natl Acad Sci USA.

[18] Driscoll WW, Travisano M (2017). Synergistic cooperation promotes multicellular performance and unicellular free-rider persistence. Nat Commun.

[19] Ratcliff WC, Fankhauser JD, Rogers DW, Greig D, Travisano M (2015). Origins of multicellular evolvability in snowflake yeast. Nat Commun.

[20] Abouheif E (2014). Eco-evo-devo: the time has come. Adv Exp Med Biol.

[21] Gilbert SF, Bosch TCG, Ledon-Rettig C (2015). Eco-Evo-Devo: developmental symbiosis and developmental plasticity as evolutionary agents. Nat Rev Genet.

[22] Khurana N, Saxena RK, Gupta R, Kuhad RC (1993). Light-independent conidiation in Trichoderma spp.: a novel approach to microcycle conidiation. World J Microbiol Biotechnol.

[23] Kindermann J, El-Ayouti Y, Samuels GJ, Kubicek CP (1998). Phylogeny of the genus trichoderma based on sequence analysis of the internal transcribed spacer region 1 of the rDNA cluster. Fungal Genet Biol.

[24] Gessner MO, Bauchrowitz MA, Escautier M (1991). Extraction and quantification of ergosterol as a measure of fungal biomass in leaf litter. Microb Ecol.

[25] Kim D (2013). TopHat2: accurate alignment of transcriptomes in the presence of insertions, deletions and gene fusions. Genome Biol.

[26] Trapnell C (2012). Differential gene and transcript expression analysis of RNA-seq experiments with TopHat and Cufflinks. Nat Protoc.

[27] Szklarczyk D (2011). The STRING database in 2011: functional interaction networks of proteins, globally integrated and scored. Nucleic Acids Res.

