# Supplementary material for: Developmental evolution facilitates rapid adaptation
Source: Sci Rep. 2017 Nov 21;7:15891. doi: 10.1038/s41598-017-16229-0 (PMC5698356; doi:10.1038/s41598-017-16229-0)
Supplement: Supplementary file 1 — Supplemental Information [file 41598_2017_16229_MOESM1_ESM.pdf]

Developmental evolution facilitates rapid adaptation

Hui Lin[1], Romas J. Kazlauskas[1,2], and Michael Travisano[1,3]

[1]BioTechnology Institute, University of Minnesota, St Paul, Minnesota 55108, USA

[2]Department of Biochemistry, Molecular Biology and Biophysics, University of Minnesota, St Paul, Minnesota 55108, USA

[3]Department of Ecology, Evolution and Behavior, University of Minnesota, St Paul, Minnesota 55108, USA.

Author for correspondence:
Michael Travisano,
travisan@umn.edu

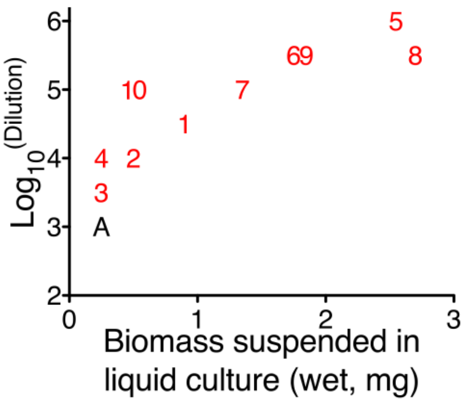

Supplementary Figure S1. Live particles and suspended biomass in the liquid of the ancestral (A) and 85_{th} derived (numbers) *T. citrinoviride* cultures. Y axis scale Log_{10}(Dilution) means $10^{(Y)}$ live particles in 1 mL suspension liquid. While the selected populations had more suspended biomass than the ancestor (Fig. 1b), the total biomass (Supplementary Fig. s2) was similar or less than the ancestor. Serial of dilution growth linearly related to the biomass in the supernatant, and eight of the ten derived populations have more biomass in the supernatant. All ten derived populations have more live particles in the supernatant than the ancestor.

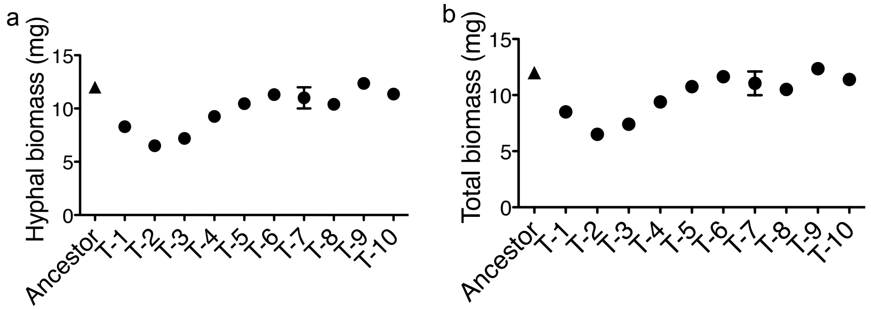

Supplementary Figure S2. Hyphal (a) or total (hyphae + conidia) (b) biomass of ancestor (A) and 85th derived populations (numbers). Only derived population 9 yielded similar amounts of hyphal and total biomass as the ancestor at 24 hours, while all the other selected populations accumulated less biomass than the ancestor, especially populations 2 and 3, which yielded only 50% of the biomass of the ancestor.

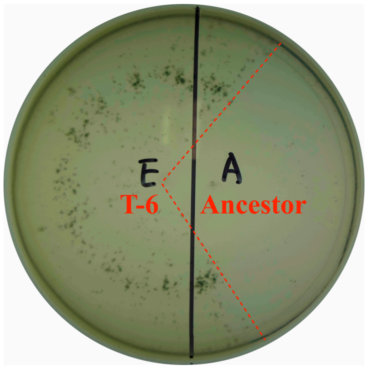

Supplementary Figure S3. The solid culture of T-6 and ancestor on malt extract agar media. The evolved T-6 also grew faster, and produced conidia earlier that the ancestor.

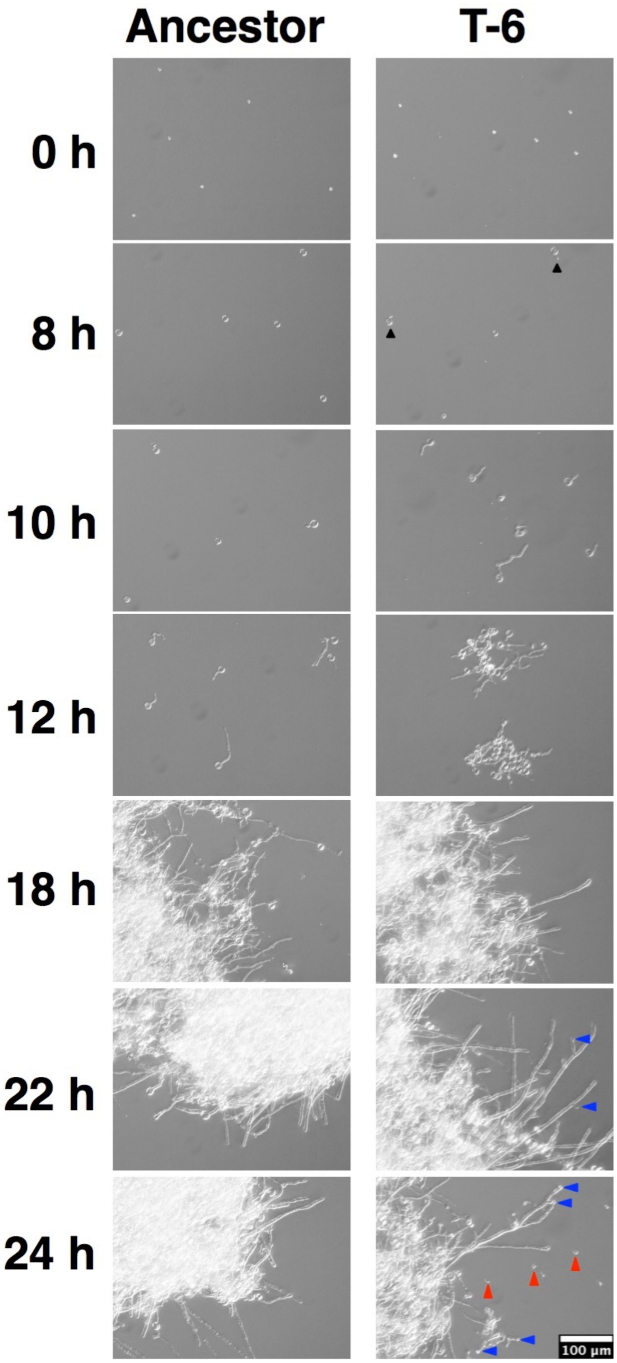

Supplementary Figure S4. Ancestor versus T-6 life cycle under microscope. (1) T-6 conidia started germination (Black triangle) at 8 hours, while no ancestral conidia germination within 8 hours; (2) Few ancestor conidia germinated after 10 hours, while over 50% of the T-6 conidia germinated within 10 hours; (3) Both ancestor and T-6 formed mycelium pellets, no conidiaphore development was found in the ancestor cultures after 22 hours, while many conidiaphores (Blue triangle) were detected in T-6 cultures. (4) The ancestor did not produce conidia (Red triangle) by 24 hours, while T-6 produced large numbers of conidia (about 10^6 conidia per mL).

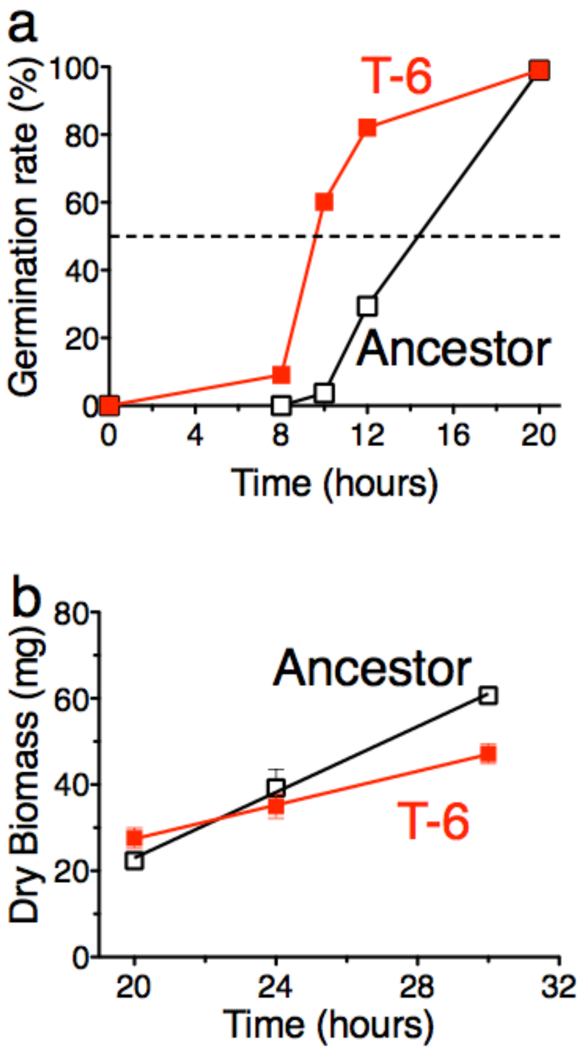

Supplementary Figure S5. Conidia germination (*a*) and biomass accumulation (*b*) of ancestor (open square) and T-6 (solid square) in malt extract media. (*a*) T-6 took about nine hours for 50% germination, while the ancestor took 15 hours to achieve 50% germination. After 20 hours, ≥99% of the conidia germinated in both ancestral and T-6 populations. (*b*) T-6 accumulated about 20% more hyphae biomass than ancestor at 20 hours, while ancestor accumulated 11% and 29% more hyphae biomass than T-6 at 24 and 30 hours respectively.

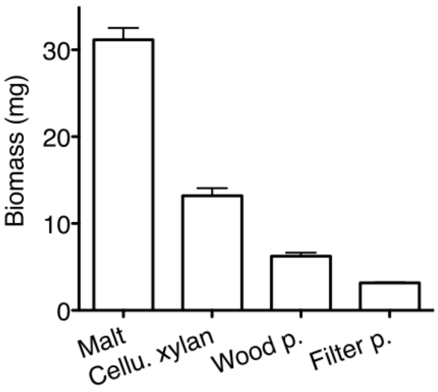

Supplementary Figure S6. Mycelial growth of ancestral *T. citrinoviride* in different media. Cellulose and xylan media, wood powder media and filter paper media were used to support the conidiation of *T. citrinoviride* over a four-fold range of accumulated mycelia.

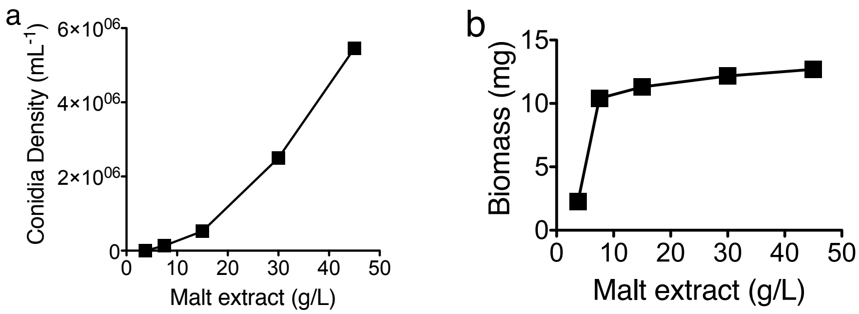

Supplementary Figure S7. Conidia and biomass of T-6 across a gradient of malt extract media. Conidia production increases with more malt extract, at approximately 2 x 10^5 conidia per g/L of malt extract in the growth media. There are no conidia produced at the lowest concentrations of malt extract, 3.75 g/L. In contrast, additional malt extract only modestly increases biomass, except at the lowest concentrations. At the lowest concentration, 3.75 g/L malt extract supplemented media, there is poor spore germination leading to poor biomass production over the course of the experiment. While there is better germination with higher concentrations of malt extract, there is little effect on biomass.

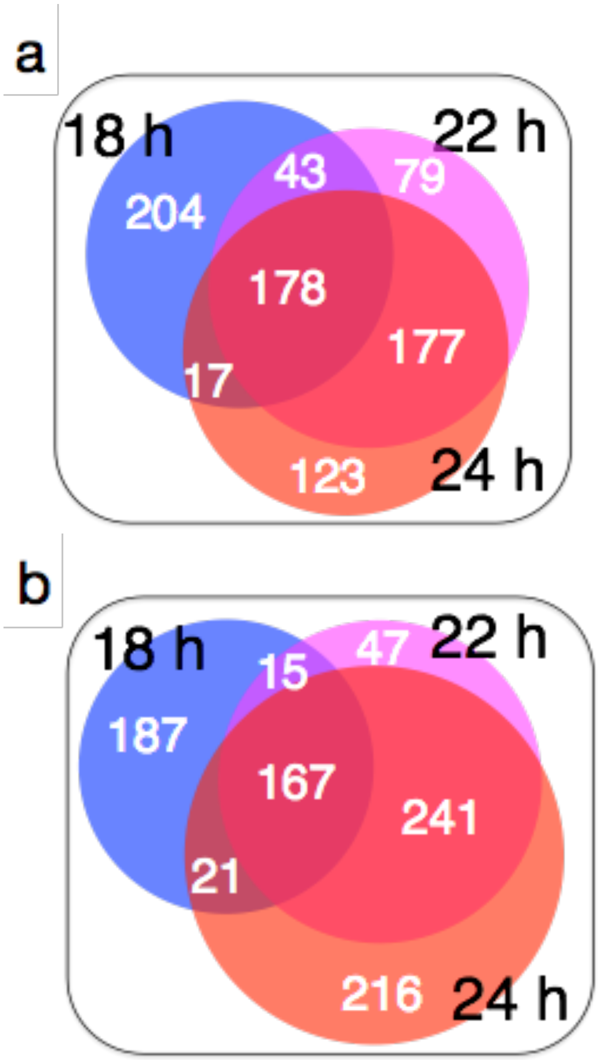

Supplementary Figure S8. Transcription of ancestor and T-6 in malt extract liquid cultures. (*a*) Venn diagram of 821 down-regulated loci in T-6. (*b*) Venn diagram of 894 up-regulated loci in T-6. 20% of both down regulated and up regulated loci in T-6 existed at 18, 22, and 24 hours, which did not relate to temporal development. Fewer overlap loci only existed in 18 and 22 hours (43-down or 15-up loci), or 18 and 24 hours (17-down or 21-up loci) than the overlaps in 22 and 24 hours (177-down and 241-up), indicating the big temporal development from 22 to 24 hours.

Table S1, The transcriptional levels of wetA and abaA in ancestor and T-6 in malt extract media.

Loci	Time (hour)	log2(fold-change), ancestor/T-6
wetA	18	-1.89
	22	-3.23
	24	-4.28
abaA	18	-0.81
	22	-3.67
	24	-4.59
